# Alpha-Fetoprotein-Producing Gastric Cancer With Delayed Diagnosis Caused by COVID-19: A Case Report

**DOI:** 10.7759/cureus.27392

**Published:** 2022-07-28

**Authors:** Satoshi Masuyama, Mimari Kanazawa, Keiichi Tominaga, Kazuyuki Ishida, Atsushi Irisawa

**Affiliations:** 1 Department of Gastroenterology, Dokkyo Medical University, Mibu, JPN; 2 Department of Diagnostic Pathology, Dokkyo Medical University, Mibu, JPN

**Keywords:** delayed diagnosis, upper gastrointestinal bleeding, alpha-fetoprotein-producing gastric cancer, urgent esophagogastroduodenoscopy, covid-19

## Abstract

A 70-year-old man was diagnosed with coronavirus disease 2019 (COVID-19). The patient had suspected upper gastrointestinal bleeding during the course of the COVID-19 infection. Urgent esophagogastroduodenoscopy (EGD) was performed. However, because of mobility restrictions imposed as a COVID-19 countermeasure, EGD was done in a small hospital room. Hemostatic treatment was successful, but no sufficient close examination could be done. The patient, who was diagnosed as having alpha-fetoprotein-producing gastric cancer, died about three months later.

## Introduction

The coronavirus disease 2019 (COVID-19) has spread quickly to become a global pandemic. Because exposure to droplets and aerosols from coughing induced by gastrointestinal endoscopy is believed to increase the risk of severe acute respiratory syndrome coronavirus 2 (SARS-CoV-2) infection, adequate infection control measures must be used when testing COVID-19 patients [1,2]. Moreover, the Japan Gastroenterological Endoscopy Society (JGES) recommends that endoscopy for COVID-19 patients be postponed, except for emergency procedures [3]. Previous reports have described that under appropriate triage, delayed gastrointestinal endoscopy is unlikely to affect prognosis significantly [4]. However, a large institutional report in the United Kingdom described delayed diagnosis of gastrointestinal malignancies in 37 out of 1 million patients per month [5].

This report describes a case of alpha-fetoprotein (AFP)-producing gastric cancer in which COVID-19 infection in a patient delayed definitive diagnosis by gastrointestinal endoscopy. Furthermore, we discuss the appropriate triage and endoscopic procedures used during the COVID-19 pandemic.

## Case presentation

A 70-year-old man visited his local doctor in November 2020 because of fever of 37.8°C, malaise, sore throat, and dyspnea. The patient was diagnosed with COVID-19 based on polymerase chain reaction (PCR) testing for SARS-CoV-2. Chest computed tomography (CT) showed a ground-glass shadow on the periphery of the bilateral lower lung fields. The patient was referred to our hospital for treatment and was hospitalized. The patient has type 2 diabetes, dyslipidemia, and a history of colon cancer surgery at age 55. The patient was an occasional drinker and had smoked 80 cigarettes/day for 28 years (age 19-47). The patient had no previous allergy or familial history.

**Figure 1:**
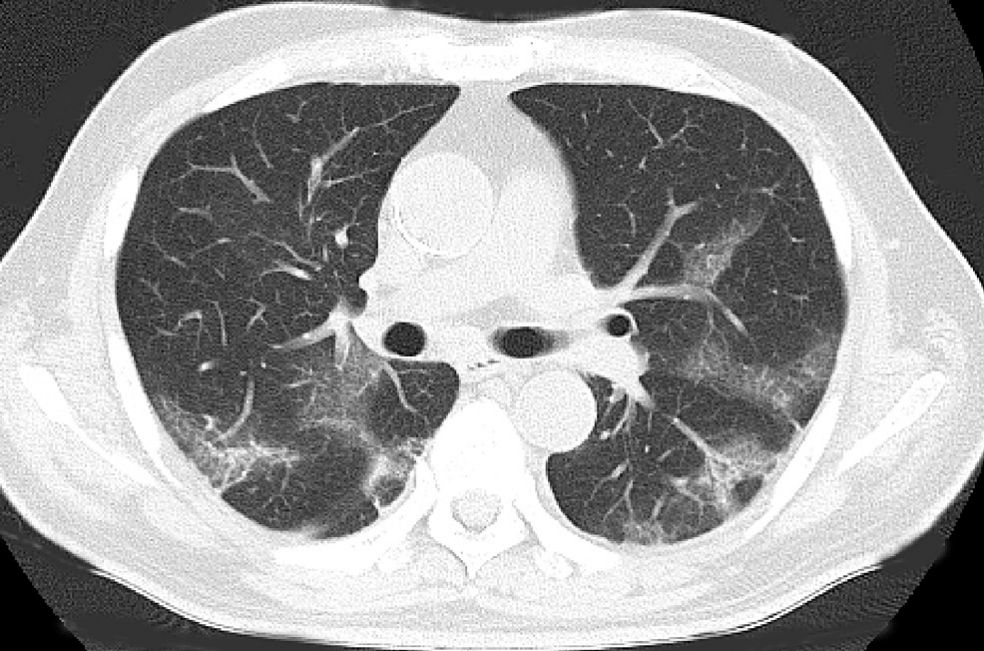
Chest computed tomography showing subpleural ground-glass shadows in the bilateral lower lobes. Typical image of acute interstitial pneumonia as coronavirus disease 2019.

**Figure 2:**
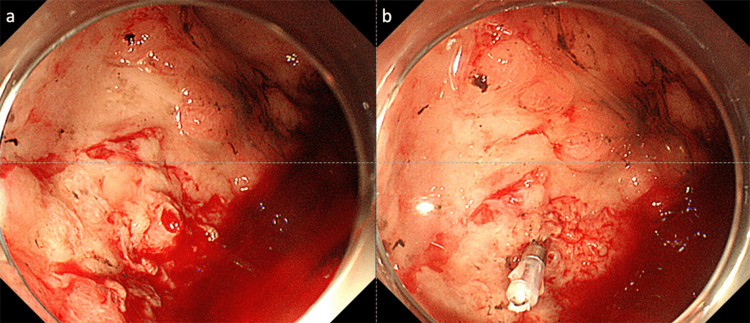
Esophagogastroduodenoscopy showing a widespread ulcer with an exposed vessel in the gastric fornix (a). Therefore, endoscopic hemostasis was performed using hemoclips (b).

Laboratory data obtained at admission showed no liver or renal dysfunction. White blood cell count was 4,000/μL, neutrophil 80.1%; C-reactive protein was 4.31 mg/dL. Anemia was detected, with hemoglobin (Hb) of 10.9 g/dL (Table 1). Chest CT showed subpleural ground-glass shadows in the bilateral lower lobes and a typical image of acute interstitial pneumonia as COVID-19 (Figure 1). On admission, SpO_2_ was less than 90% under nasal oxygen cannula administration at 1 L/min. The disease severity of COVID-19 by the World Health Organization (WHO) was considered severe. Treatment of COVID-19 was initiated with favipiravir and methylprednisolone. On the 15th hospital day, melena was observed. His blood pressure had fallen to 88/47 mmHg, which was thought to be caused by hemorrhagic shock. Laboratory data showed Hb 4.8 g/dL, blood urea nitrogen (BUN) 75.4 mg/dL, and creatinine (Cre) 1.04 mg/dL. Upper gastrointestinal bleeding was suspected. Urgent esophagogastroduodenoscopy (EGD) was considered necessary. However, because of mobility restrictions imposed by COVID-19 countermeasures, the patient was not allowed to be examined in the endoscopy room. Instead, EGD was done in a small hospital room. Staff involved in EGD were kept to a minimum. Personal protective equipment (PPE) recommended by the JGES was used thoroughly during the endoscopic examination. Detailed observation was difficult because the patient’s blood pressure was unstable during the endoscopy. Because endoscopic examination revealed a widespread ulcer with an exposed vessel in the posterior wall of the gastric fornix, endoscopic hemostasis was performed using hemoclips. The procedure was completed within 25 minutes (Figures 2a, 2b). Subsequently, vonoprazan was administered. Fortunately, pneumonia caused by COVID-19 improved. The patient was discharged on the 27th hospital day because the criteria for discharge set by the Japanese Ministry of Health, Labour and Welfare were met. After endoscopic treatment, his anemia did not progress until the day of discharge. It was determined that hemostasis had been achieved. Hb was 7.9 g/dL at discharge.

**Table 1:** Laboratory data on the first admission. WBC: white blood cell; RBC: red blood cell; AST: aspartate transaminase; ALT: alanine transaminase; GGT: gamma-glutamyl transferase; CRP: C-reactive protein; HBsAg: hepatitis B surface antigen; HCV: hepatitis C virus; CMV: cytomegalovirus; SP-D: surfactant protein-D

Complete blood count	Value	Unit	Normal range	Chemistry	Value	Unit	Normal range	Serology	Value	Unit	Normal range
WBC	4,000	/μL	4000–9,000	AST	32	U/L	13–30	KL-6	204	U/mL	<500
RBC	3.49	10^12^/L	1.3–5.7	ALT	19	U/L	10–42	HBs-Ab	Negative		
Hemoglobulin	10.9	g/dL	13.5–17.5	ALP	187	U/L	38–113	HCV-Ab	Negative		
Hematocrit	33.8	%	40–53	Lactate dehydrogenase	279	U/L	124–222	β-D-glucan	4.7	pg/mL	<20.0
Platelet	13	10^9^/L	15–35	GGT	14	IU/L	13–64	Influenza test	A-B-		
				Total bilirubin	0.4	mg/dL	0.4–1.5	*Streptococcus pneumoniae* antigen	Negative		
Neutrophil	80.1	%	40.0–69.0	Albumin	2.9	g/dL	4.0–5.1	Urinary antigen of *Legionella*	Negative		
Eosinophil	0	%	0.0–5.0	Urea nitrogen	19.6	mg/dL	8.0–20	Ferritin	130.2	ng/mL	17.0–291.5
Basophil	0	%	0.0–2.0	Na	130	mmol/L	138–145	Procalcitonin	0.04	ng/mL	<0.05
MoC	5.3	%	3.0–9.0	K	3.6	mmol/L	3.6–4.8	CMV-Ag (C10C11)	Negative		
Lymphocyte	14.6	%	26.0–46.0	Cl	102	mmol/L	101–108	SP-D	56.6	ng/mL	<110
				Creatinine	1.00	mg/dL	0.65–1.07	*Aspergillus *antigen	Negative		
				CRP	4.31	mg/dL	<0.01	*Cryptococcus neoformans* antigen	Negative		
				ESR (1 hour)	40	mm	2.0-10	T-SPOT	Negative		
				HbA1c	8.7	%	4.6-6.2				

**Figure 3:**
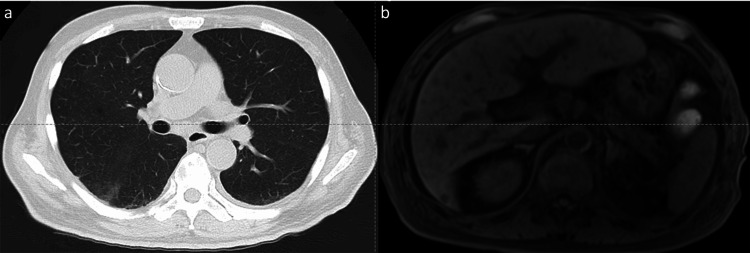
Chest computed tomography showing improvement of pneumonia (a). Abdominal gadolinium-ethoxybenzyl-diethylenetriamine pentaacetic acid-enhanced magnetic resonance imaging (hepatocyte phase) showing multiple tumors in the liver (b). Multiple tumors with ring-shaped contrast effect without early stain, and the lesion is unlikely to be hepatocellular carcinoma.

**Figure 4:**
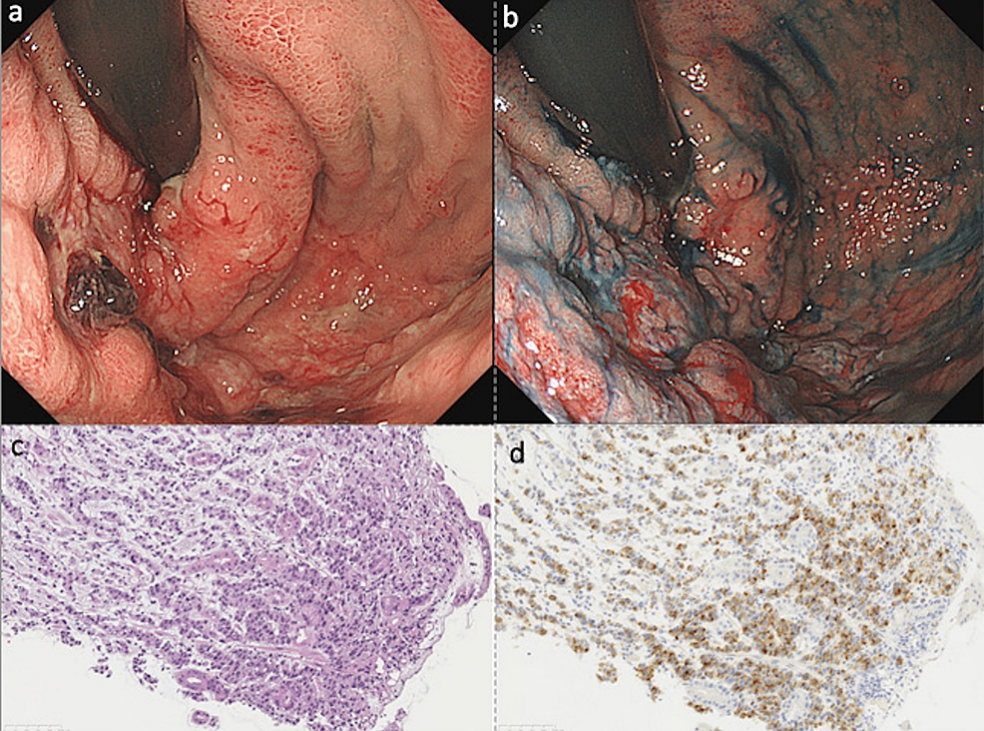
Esophagogastroduodenoscopy showing diffuse, borderless, irregularly shaped ulcers from the esophagogastric junction to the fornix and corpus of the stomach (a and b). Biopsy specimen from the edge of the ulcer diagnosed poorly differentiated adenocarcinoma (c, hematoxylin and eosin staining ×200). Alpha-fetoprotein immunohistochemical staining was positive (d, ×200).

Thereafter, no gastrointestinal bleeding occurred. However, three months after discharge from the hospital, the patient was examined by a local physician when he became aware of fatigue. The patient again consulted our hospital because he was found to be anemic. Laboratory data showed anemia with Hb 8.0 g/dL. Chest CT revealed improving pneumonia, but abdominal magnetic resonance imaging (MRI) showed multiple tumors in the liver (Figures 3a, 3b). Peritoneal dissemination and para-aortic lymph node metastasis were also observed. Tumor markers were elevated at carcinoembryonic antigen (CEA) 2,334 ng/mL and AFP 8,103 ng/mL (Table 2). Because the previous endoscopy was performed emergently under COVID-19 restrictions, the ulcer had been only inadequately observed because of bleeding. However, detailed observation was possible during the present EGD. The EGD showed diffuse, borderless, irregularly shaped ulcers from the esophagogastric junction to the fornix and corpus of the stomach (Figures 4a, 4b). A biopsy specimen from an irregular mucosal edge of the ulcer was diagnosed as poorly differentiated adenocarcinoma (Figure 4c). Furthermore, immunohistochemical staining of pathology specimens showed that approximately 20% of tumor cells were positive for AFP (Figure 4d), indicating that the diagnostic criteria for AFP-producing gastric cancer were satisfied. Thereafter, his general condition deteriorated rapidly. The patient died on the 11th hospital day during the second admission because of cachexia related to the cancer progression.

**Table 2:** Laboratory data on the second admission. WBC: white blood cell; RBC: red blood cell; AST: aspartate transaminase; ALT: alanine transaminase; GGT: gamma-glutamyl transferase; CRP: C-reactive protein; CEA: carcinoembryonic antigen; CA-19-9: cancer antigen-19-9; AFP: alpha-fetoprotein; PIVKA II: protein induced by vitamin K absence-II

Complete blood count	Value	Unit	Normal range	Chemistry	Value	Unit	Normal range
WBC	7,500	/μL	4,000–9000	AST	251	U/L	13–30
RBC	2.94	10^12^/L	1.3–5.7	ALT	151	U/L	10–42
Hemoglobulin	8.0	g/dL	13.5–17.5	ALP	471	U/L	38–113
Hematocrit	25.5	%	40–53	Lactate dehydrogenase	638	U/L	124–222
Platelet	25	10^9^/L	15–35	GGT	58	IU/L	13–64
				Total bilirubin	0.9	mg/dL	0.4–1.5
Neutrophil	66.1	%	40.0–69.0	Albumin	3.2	g/dL	4.0–5.1
Eosinophil	1.2	%	0.0–5.0	Urea nitrogen	30.2	mg/dL	8.0–20
Basophil	0.8	%	0.0–2.0	Na	130	mmol/L	138–145
MoC	9.1	%	3.0–9.0	K	4.1	mmol/L	3.6–4.8
Lymphocyte	22.8	%	26.0–46.0	Cl	96	mmol/L	101–108
				Creatinine	0.92	mg/dL	0.65–1.07
				CRP	5.23	mg/dL	<0.01
				CEA	2334.35	ng/mL	<5.0
				CA-19-9	9.97	U/mL	<37.0
				AFP	8103.3	ng/mL	<20.0
				PIVKA II	36	mAU/mL	<40.0

## Discussion

The presented case was of AFP-producing gastric cancer for which endoscopic re-examination was not performed because of COVID-19, resulting in a delay in making the definitive diagnosis. Usually, when an ulcer lesion is found during emergency EGD, second-look EGD is performed within a few days to confirm hemostasis of the lesion and perform a biopsy for malignant potential. However, because the patient was isolated for treatment of COVID-19, it was difficult to schedule the second-look EGD. In Japan, the JGES recommends that gastrointestinal endoscopy during the COVID-19 pandemic be postponed as long as possible, except in emergency cases, considering the risk of infection [3,6-8]. In this case, the patient’s vital signs were stable after the emergency endoscopy. There was no worsening of anemia. Therefore, we considered that hemostasis had been achieved. In consideration of the infection risk described above, it was determined that the second-look EGD was not indicated and that it should be postponed because the patient was at high risk of SARS-CoV-2 infection. Furthermore, the SARS-CoV-2 PCR test at discharge was positive. We assessed that it would be difficult to perform the early second-look EGD after discharge from the hospital. However, we hypothesized that this decision delayed the definitive diagnosis and that it led to an early mortality outcome. In performing endoscopy during the COVID-19 pandemic, the symptomatic patient caused by SARS-CoV-2 infection was considered cured if 10 days had passed since the onset date and 72 hours had elapsed since the relief of symptoms. Or even if 10 days had not elapsed, if two times PCR tests were performed at least 24 hours apart after symptoms had abated and a negative result was confirmed, the patient was considered cured and endoscopy could be performed as usual. In this case, the patient met the above criteria, and a reexamination should have been scheduled as soon as possible after discharge from the hospital. However, because the patient had a positive SARS-CoV-2 PCR test at the time of discharge, there was a risk of recurrence of COVID-19 even after discharge. Furthermore, there were various restrictions on endoscopy, including the infection control measures at our facility and the patient’s life background, which limited the ability to perform endoscopy.

Gastrointestinal endoscopy might induce coughing. Some concern exists about airborne infection by aerosolized SARS-CoV-2 in addition to droplet and contact infection [9]. Reportedly, SARS-CoV-2 can survive for several hours in the air [10]. Therefore, exposure to highly contaminated aerosols for a certain period of time in an enclosed space such as an endoscopy room is expected to lead to a high frequency of viral infections [1,2]. The infection rate among healthcare workers working in an endoscopy section is reportedly 4.66 times higher than among other healthcare workers [11]. Furthermore, in an endoscopy room with non-negative pressure and limited ventilation, it is difficult to perform gastrointestinal endoscopy if the SARS-CoV-2 PCR test is positive, even if the patient satisfies the discharge criteria. Nevertheless, the possibility of infection from patients to healthcare workers is reported to be low if the worker’s PPE is maintained adequately [12].

Reportedly, the COVID-19 pandemic has had numerous and diverse effects on cancer care by limiting testing and deterring physicians’ consultations with patients. Gastric cancer is the second leading cause of cancer death in Japan in 2019 for men and fourth for women. Early diagnosis of gastric cancer by EGD has important benefits for prognosis. Several reports have indicated that COVID-19 has decreased the number of gastrointestinal endoscopies performed and has thereby reduced the rate of diagnosis. A report from Italy showed a 15.9% decrease in the number of gastric cancer cases diagnosed in 2020 compared to those in 2019 [13]. In addition, a large study in the United Kingdom reported a delayed diagnosis of digestive malignancies in 37 patients per million population per month [5]. Although the diagnosis of this case was delayed because of various circumstances associated with COVID-19, the AFP-producing gastric cancer might also have contributed to this unfortunate outcome. In fact, AFP-producing gastric cancer has a rapid cell proliferation rate. The five-year survival rate is approximately 22%, which is significantly lower than that for usual gastric cancer. In addition, it was reported that 23% of patients with AFP-producing gastric cancer could be diagnosed at stage I or II when curative resection is possible [14]. Because of the high rates of liver and lymph node metastasis, AFP-producing gastric cancer is regarded as a high-grade gastric cancer with a poor prognosis. A delay in diagnosis can strongly affect unfortunate prognoses.

## Conclusions

During the COVID-19 pandemic, gastrointestinal endoscopy has often been restricted. However, when performing endoscopies, endoscopists should always be reminded to minimize the influence of COVID-19 on the underlying disease by appropriately considering necessary environmental arrangements and firm protective measures.
